# The effect of aging on genetic parameters of boar semen traits

**DOI:** 10.1093/jas/skaf257

**Published:** 2025-08-01

**Authors:** Pedro Sá, Rodrigo M Godinho, Marta Gòdia, Claudia A Sevillano, Barbara Harlizius, Ole Madsen, Henk Bovenhuis

**Affiliations:** Wageningen University & Research, Department of Animal Sciences, Gelderland, The Netherlands; Topigs Norsvin Research Center B.V., North Brabant, The Netherlands; Wageningen University & Research, Department of Animal Sciences, Gelderland, The Netherlands; Topigs Norsvin Research Center B.V., North Brabant, The Netherlands; Topigs Norsvin Research Center B.V., North Brabant, The Netherlands; Wageningen University & Research, Department of Animal Sciences, Gelderland, The Netherlands; Wageningen University & Research, Department of Animal Sciences, Gelderland, The Netherlands

**Keywords:** aging, genetic parameters, pig, semen traits

## Abstract

The main objective of this study was to investigate whether the genetic parameters of porcine semen traits change with the age of the boar. The dataset included records from 449,966 ejaculates collected for artificial insemination from 5,692 boars from a commercial line between 7 and 60 mo of age. These records included 12 semen quantity and quality traits measured with a Computer-Assisted Sperm Analysis system. First, an animal repeatability model was applied to the complete dataset to predict the effect of the age of the boar at collection on semen traits. Based on the predicted effects of age, the dataset was divided into 3 age classes: 7 to 13 mo, 14 to 23 mo, and 24 to 60 mo. Traits measured in these age classes were analyzed using a trivariate model to estimate variance components and genetic correlations between traits at different ages. The unadjusted raw averages of semen quality traits with age differed considerably from the model predictions, likely because boars with low semen quality were culled at younger ages and were absent from the data at older ages. Model predictions showed that semen quantity traits increased early in production and plateaued around 18 mo, while sperm motility peaked at 13 mo and gradually declined. Sperm morphology was lowest at 11 mo and gradually increased after. Genetic correlations between the traits at different age classes were close to unity, indicating that, genetically, semen traits at different ages are identical traits. Heritabilities were stable across age classes for all semen traits. Additive genetic variances remained stable for semen quality traits with age class but increased for semen quantity, indicating that the effects of genetic variants affecting semen quantity traits increase with the age of the boar.

## Teaser

This study describes changes in the genetics of male reproductive traits in pigs as boars age.

## Introduction

In mammalian species, it is generally known that semen quality declines with age (Schwartz et al., 1983; Kidd et al., 2001; Salimiyekta et al., 2023). In pigs, an increase in sperm abnormalities and a decline in sperm motility with the boar’s age have been observed (Oh et al., 2006; Wolf and Smital, 2009; Strathe et al., 2013). The reproductive performance of boars plays a crucial role in the effective transfer of genetic progress within pure lines and to commercial crossbred pigs. Despite compelling evidence of phenotypic changes in the reproductive performance of males with age, less is known about how the genetics of these traits change with age. Most studies on porcine semen traits used animal repeatability models, assuming that traits remain genetically the same throughout the production period of boars (Marques et al., 2017; Ogawa et al., 2022; Sá et al., 2025). Some studies have used random regression models, allowing the genetic background of semen traits to change with age, and reported conflicting results. Strathe et al. (2013) reported strong positive genetic correlations between semen traits measured at different ages, whereas Oh et al. (2006) and Hong et al. (2022) found weak or even negative genetic correlations. These inconsistent observations require further analysis to clarify the relationship between the genetic background of semen traits and age. This is essential for advancing our biological understanding of these traits and for selecting the appropriate statistical model when analyzing semen traits for breeding value estimation and genome-wide association studies or examining characteristics derived from semen samples, such as RNA-seq or methylation patterns (Gòdia et al., 2020).

In this study, we aim to determine if the genetics of semen traits change with the age of the boar at collection. We examined semen records measured throughout the production period of boars from a commercial line and addressed whether the genetics of these traits change with the boar’s age. First, we provide a phenotypic description of the effect of the boar’s age on semen traits. For the genetic analyses, we divide records into 3 different age classes based on the age of the boar at collection to estimate: i) changes in variance components, heritabilities, and repeatabilities of semen traits with age and ii) genetic correlations between semen traits at different age classes.

## Materials and Methods

### Ethics statement

The data used in this study came from routine semen collections from pigs of a commercial pig line. Sample collection and data recording complied with Dutch animal protection laws (Gezondheids- en welzijnswet voor dieren) to ensure ethical standards.

### Phenotypes

Fresh ejaculates were collected from boars of a Topigs Norsvin commercial Large White line between January 2009 and January 2022. These boars were used commercially for artificial insemination (AI), and a considerable number of inseminations worldwide are performed with semen from these boars to produce growing-finishing pigs. The ejaculates were collected using a semi-automated collection system and immediately pre-diluted 1:1 with Solusem Bio + (IMV). The pre-diluted fresh semen was analyzed using an IVOS Computer-Assisted Sperm Analysis (CASA) system (Hamilton Thorne Inc., Beverly, MA). In addition, data on microscopy assessments of head morphological abnormalities were available. The microscopy measurements were conducted by experienced technicians and included staining of an ejaculate smear and identifying and counting sperm cells with abnormal head morphology. After the evaluation of fresh semen using CASA and microscopy, the ejaculates were fully diluted to a final dose concentration of 1.3 to 1.5 billion sperm cells and stored at 17 °C in commercial doses of 80 mL. After 3 d of storage, a random subset of these commercial doses were evaluated again with the CASA system.

The dataset included records on semen quantity traits (i.e., total number of sperm cells, ejaculate volume, and concentration of sperm cells) and semen quality traits (i.e., total and progressive motility of fresh semen as well as total and individual morphological abnormalities). The latter included CASA records of distal cytoplasmatic droplets, distal midpiece reflex and bent tails, and microscopy records of head morphological abnormalities. The semen assessment after 3 d of storage included CASA measurements of total and progressive motility traits after storage. All motility and morphology traits were expressed in percentages. The complete dataset included phenotypes from 449,966 ejaculates collected on 5,692 boars, with an average of 79 ejaculates per boar and 5th and 95th percentiles of 7 and 202 ejaculates, respectively.

### Statistical analyses

Model [1] describes the animal repeatability model used to predict the effect of the age of the boar at collection on semen traits:


$$\begin{array}{l} y=X\beta +Za+Wp+Hh+Cc+\;e \end{array}$$ [1]

***y*** represents a vector with phenotypic observations of semen traits; **X** is the incidence matrix of fixed effects of age of the boar at collection, 54 classes of 1 mo each; the number of days the boar rested since the last collection, 15 classes of 1 d each; the mo of collection, 12 classes in months of the year; **Z**, **W**, **H** and **C** are incidence matrices for the random additive genetic, permanent environment, and herd-year-season of birth of the boar and collector-lab technician effects, respectively; β, a, p, h and c are the vectors with solutions for fixed (β) and random effects (a, p, h  and c). Random effects are assumed to be distributed as $a∼N\left(0,\;\;\;A\sigma_{a}^{2}\right)$ where A is the pedigree-based relationship matrix and $\;\;\sigma_{a}^{2}$ is the additive genetic variance, $p∼N\left(0,\;\;\;I\sigma_{pe}^{2}\right)$ where I is the identity matrix, $\sigma_{pe}^{2}$ is the permanent environment variance, $h∼N\left(0,\;\;\;I\sigma_{hys}^{2}\right)$, where I is the identity matrix and $\sigma_{hys}^{2}$, the variance of herd-year-season of birth of the boar, $c∼N\left(0,\;\;\;I\sigma_{clt}^{2}\right)$, where I is the identity matrix and $\sigma_{clt}^{2}$, the variance of collector-lab technician and $e∼N\left(0,\;\;\;I\sigma_{e}^{2}\right)$, where I is the identity matrix and $\sigma_{e}^{2}\;\;\;$ is the variance of residual effects.

Analyses were performed using ASREML v.4.2 (Gilmour et al., 2021). The **A** matrix was constructed based on pedigree information from 26 generations and included 17,701 individuals. For 90% of the boars, all ancestors in the pedigree up to 8 generations back were known.

The age of the boars at collection ranged from 7 to 60 mo. The effect of the age of the boar was predicted by averaging the phenotype over the effect of all the other terms in the model using the *“predict”* statement in ASREML. The distributions of motility traits were right-skewed and the distributions of morphology traits were left-skewed. In preliminary models for sperm motility and morphology traits, the residuals appeared approximately normal, but plots of fitted versus residual values showed heteroscedasticity, indicating non-constant variance. To meet model assumptions of normality of residuals and homogeneity of variance, we applied trait transformations to stabilize variance and improve model fit. We applied a cubic transformation to sperm motility traits and a log transformation to sperm morphology traits (Osborne, 2010). For clarity of interpretation, age effects were predicted using untransformed traits, whereas variance components, heritabilities, and repeatabilities were estimated using the transformed data.

### Trivariate analyses

In order to investigate if semen traits remain genetically the same trait throughout the full collection period of boars, we used a trivariate analysis and estimated additive genetic variances and heritabilities for different age classes and genetic correlations between the same trait measured in different ages. Records of semen traits were divided into 3 age classes: early production period (7 to 13 mo), peak production (14 to 23 mo), and post-peak production (24 to 60 mo). These age classes were determined by combining known physiological stages of boar reproductive development with the age trends predicted for semen traits using the model [1]. The early production period reflects the stage shortly after puberty, with increasing semen output and before peak production (Anderson, 2009; Kamanová et al., 2017). Peak production corresponds to the age of maximal semen quantity and quality (Anderson, 2009; Hu et al., 2024), and post-peak includes the phase of gradual reproductive decline (Tsakmakidis et al., 2012; Hu et al., 2024). For each age class, boars were included if they had at least 3 ejaculates collected during that period. Records from 61 boars were removed because these boars had less than 3 ejaculates in at least one age class.

In this population, boars whose semen was collected at 14 to 23 mo and 24 to 60 mo of age are selected subsets of boars that were collected at 7 to 13 mo of age. This selection, or culling, of boars during their collection period can be attributed to multiple factors, including their breeding value for specific traits or poor semen quality. In order to account for this selection, we assumed that boars collected at 7 to 13 mo of age were unselected for semen traits and fitted the 3 age classes in a trivariate analysis, which should account for selection as the unselected trait is included (Walter and Mao, 1985; Meyer, 1991).

This analysis was performed following an animal repeatability model similar to model [1]. In this model, age differences within each age period were accounted for with fewer classes: 7 classes for ages 7 to 13 mo, 10 classes for ages 14 to 23 mo, and 37 classes for ages 24 to 60 mo. The residuals for the same trait measured at different ages were considered independent, and the covariances were fixed to zero. Furthermore, covariances between herd-year-season of birth and collector-lab technician effects at different ages were also fixed to zero. The covariance matrices for additive genetic ($\sum_{a}$) and permanent environment ($\sum_{pe}$) effects, for the 3 ages, T1, T2 and T3, for any trait, are given as:


$$\begin{array}{l} \sum_{a}=\left[\begin{array}{lllllll} \sigma_{a_{T1}}^{2} & \sigma_{a_{T1,T2}} & \sigma_{a_{T1,T3}}\;sym & \sigma_{a_{T2}}^{2} & \sigma_{a_{T2,T3}}\;sym & sym & \sigma_{a_{T3}}^{2}\; \end{array}\right] \end{array}$$

$$\begin{array}{l} \sum_{pe}=\left[\begin{array}{lllllll} \sigma_{pe_{T1}}^{2} & \sigma_{pe_{T1,T2}} & \sigma_{pe_{T1,T3}}\;sym & \sigma_{pe_{T2}}^{2} & \sigma_{pe_{T2,T3}}\;sym & sym & \sigma_{pe_{T3}}^{2}\; \end{array}\right] \end{array}$$

The heritability (*h*^*2*^) and repeatability (*rep*) were calculated as:


$$\begin{array}{l} h^{2}=\frac{\sigma_{a}^{2}}{\sigma_{p}^{2}}\;and \end{array}$$

$$\begin{array}{l} rep=\frac{\sigma_{a}^{2}+\;\;\;\sigma_{pe}^{2}}{\sigma_{p}^{2}} \end{array}$$

$$\begin{array}{l} with\;\sigma_{p}^{2}=\sigma_{a}^{2}+\sigma_{pe}^{2}+\;\;\;\sigma_{hys}^{2}+\;\;\;\sigma_{clt}^{2}+\;\;\;\sigma_{e}^{2} \end{array}$$

The phenotypic ($r_{p_{Ti-Tj}}$), additive genetic ($r_{a_{Ti-Tj}}$), and permanent environment ($r_{pe_{Ti-Tj}}$) correlations, between any pair of ages, $T_{i}$ and $T_{j}$, for any trait, were calculated as:


$$\begin{array}{l} r_{p_{T_{i}-T_{j}}}=\frac{\sigma_{p_{T_{i}-T_{j}}}}{\sqrt{\sigma_{p_{T_{i}}}^{2}\;\;\;\sigma_{p_{T_{j}}}^{2}}} \end{array}$$

$$\begin{array}{l} r_{a_{T_{i}-T_{j}}}=\frac{\sigma_{a_{T_{i}-T_{j}}}}{\sqrt{\sigma_{a_{T_{i}}}^{2}\;\;\;\sigma_{a_{T_{j}}}^{2}}} \end{array}$$

$$\begin{array}{l} r_{pe_{T_{i}-T_{j}}}=\frac{\sigma_{pe_{T_{i}-T_{j}}}}{\sqrt{\sigma_{pe_{T_{i}}}^{2}\;\;\;\sigma_{pe_{T_{j}}}^{2}}} \end{array}$$

The estimates for these parameters were reported for transformed semen quality traits and untransformed semen quantity traits.

To determine the significance of changes in variance components, we used ASREML to calculate pairwise differences between variance components and the standard errors of these differences. We assumed the difference between 2 estimated variances followed an approximately normal distribution and the significance of these differences were tested using the Z-test. The threshold for significant differences was adjusted for multiple testing using Bonferroni. Considering a nominal significance threshold of *P*-value < 0.05 and 144 tests, the difference between 2 variances was considered significant when *P*-value < 3.5 × 10^−4^ (−log_10_(*P*-value) > 3.5).

## Results

In the results section, we begin by describing patterns in the data that reflect practical aspects of boar management. Since boars with consistently low semen quality are removed from production, we first present changes in the number of service boars in production. We then compare unadjusted means of semen traits with the age of the boar to the predicted effects estimated from model [1] to assess how observed trends relate to model-based estimates. This provides context for understanding the influence of culling on age effects. Based on the predicted age effects, we define 3 age classes to evaluate whether semen traits remain genetically consistent across different stages of boar reproductive life. In the subsequent sections, we present estimates of variance components, heritabilities, and genetic correlations across age classes.

The descriptive statistics for the complete dataset used in this study can be found in Supplementary Table 1.

### Semen traits with age of the boar at collection

**Figure 1.**
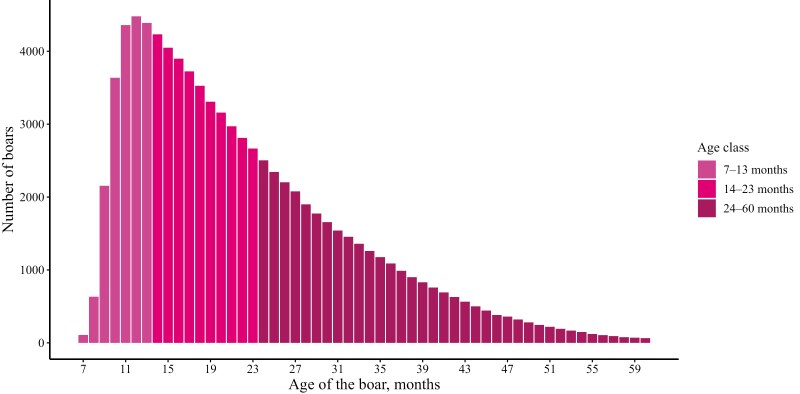
Distribution of the number of boars for semen collection by age of the boar.

Figure 1 shows the number of boars for which semen was collected as a function of their age in months. Boars were introduced into production as early as 7 mo of age, with most introduced between 9 and 10 mo, and some remained until 60 mo of age (5 yr old). The number of boars for semen collection peaked at an age between 11 and 15 mo. As boars grew older, the number of animals for semen collection continuously declined, with only a few at 60 mo of age.

**Figure 2.**
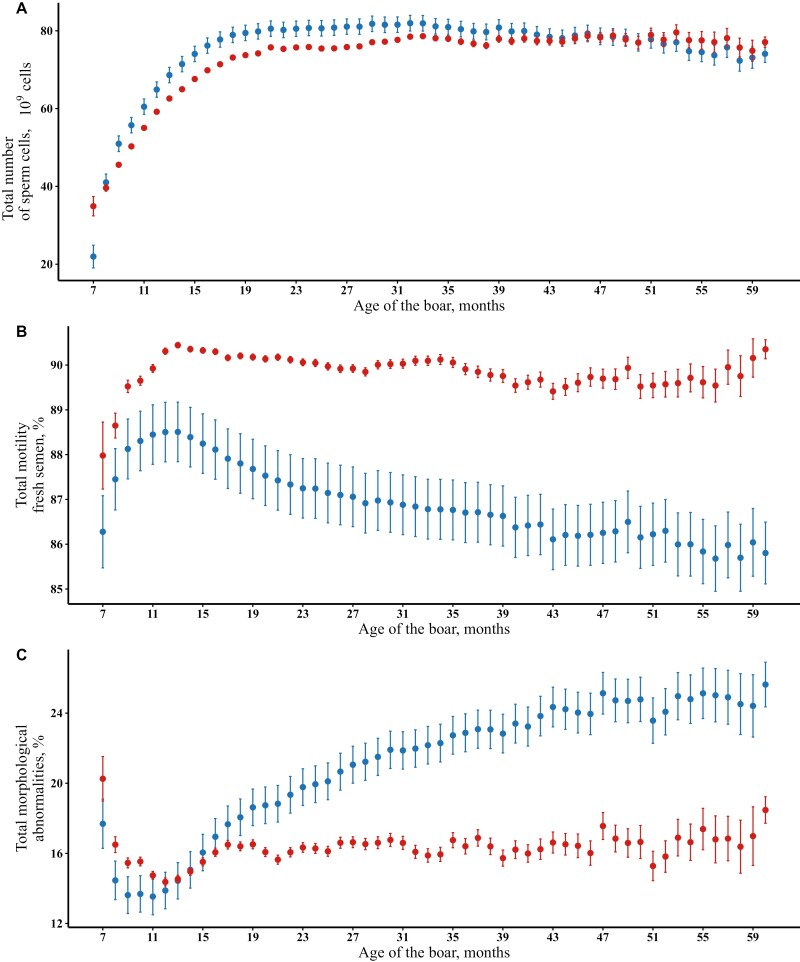
The effect of age of the boar at collection on the (**A)** total number of sperm cells in an ejaculate (in 10^9^ cells), (**B)** total motility of fresh semen (%) and (**C)** total morphological abnormalities (%). The blue markers represents the predicted effects based on model [1] and the red markers represents the unadjusted means of the trait. The error bars indicate a 95% confidence interval. Unadjusted means and estimated effects were reported for untransformed traits.

Boars with semen collections at older ages may not represent a random sample from the population, and simple trait averages at older ages might not accurately capture the true effect of age. Therefore, we compared the predicted effects with unadjusted trait means. Figure 2 shows the predicted age effects from model [1] and the mean of the unadjusted traits per month of age of the boar for the number of sperm cells in an ejaculate, total motility of fresh semen, and total morphological abnormalities. Trait averages and the predicted effects of age of the boar for all semen traits can be found in Supplementary Table 2.

For semen quantity traits (e.g., Figure 2A), predicted effects with age of the boar were similar to unadjusted trait means throughout the entire period of observation. In general, semen quantity traits increased rapidly during the early months and plateaued around 18 mo of age. For example, the lowest predicted and raw average for total number of sperm cells per ejaculate was at 7 mo followed by a steep increase until 18 mo of age. The predicted number of sperm cells plateaued between 18 and 39 mo of age. After 39 mo of age, the predicted effects slightly declined. These observations were consistent for ejaculate volume and concentration.

For sperm motility traits (e.g., Figure 2B), the predicted percentage of motile sperm cells increased during the early production period (7 to 13 mo) but declined sharply between 14 and 23 mo, followed by a more gradual decrease until 60 mo of age. The unadjusted means also increased between 7 and 13 mo of age but showed little to no change during the remaining age period. Moreover, unadjusted means of motility (red markers) were consistently higher than the predicted values (blue markers) throughout the entire period of observation. These observations were consistent for the other motility traits.

For sperm morphology traits (Figure 2C), the predicted age effects were similar to unadjusted means during the early months of collection but increasingly differed in the later period of production. The predicted rate of morphological abnormalities decreased in the first months, between 7 and 11 mo of age of the boars, and the unadjusted trait means changed accordingly. After this period, the predicted abnormality rate increased sharply between 11 and 22 mo and gradually increased until 60 mo of age (blue markers). However, these changes were not reflected in the unadjusted trait means which remained low (red markers) for the remaining period of observation. These observations were consistent for the other sperm morphology traits.

The predicted effects of the age of the boar on semen traits were used to divide semen records into 3 age classes. The classification was based on physiological and reproductive patterns: early production (adolescence and initial maturity; 7 to 13 mo), peak production (14 to 23 mo), and later production (post-peak decline; 24 to 60 mo). A description of the dataset following its division can be found in Table 1.

**Table 1.** Number of boars and number of observations, mean and standard deviation (SD) for semen traits measured during the 3 age classes

Trait^1^^)^	Age of the boar, mo	Number of boars	Observations	Mean	SD
**Semen quantity**					
**Volume, mL**	7 to 13	5,039	91,812	334.0	99.4
	14 to 23	4,340	186,589	391.5	113.2
	24 to 60	2,701	169,772	422.9	126.9
**Concentration, 10** ^**6**^ **cells/mL**	7 to 13	5,022	92,043	173.4	66.5
	14 to 23	4,345	186,426	191.4	74.9
	24 to 60	2,701	169,479	192.6	81.9
**Total number of sperm cells, 10** ^**9**^ **cells**	7 to 13	5,038	91,874	56.1	21.2
	14 to 23	4,341	186,628	71.7	25.6
	24 to 60	2,701	169,766	77.0	27.7
**Sperm motility, %**					
**Total motility of fresh semen**	7 to 13	4,999	87,764	90.0	5.6
	14 to 23	4,308	177,269	90.2	4.5
	24 to 60	2,675	160,497	89.9	4.6
**Total motility after 3 d of storage**	7 to 13	4,086	38,907	80.3	11.1
	14 to 23	3,733	78,248	79.8	10.3
	24 to 60	2,325	72,387	79.3	10.5
**Progressive motility of fresh semen**	7 to 13	5,036	91,752	80.9	9.2
	14 to 23	4,333	185,281	81.2	7.9
	24 to 60	2,696	168,392	80.6	7.9
**Progressive motility after 3 d of storage**	7 to 13	4,087	38,946	71.4	11.8
	14 to 23	3,733	78,334	71.0	11.1
	24 to 60	2,327	72,477	70.5	11.4
**Sperm morphology, %**					
**Total morphological abnormalities**	7 to 13	4,661	43,774	15.0	11.1
	14 to 23	4,155	72,344	16.0	10.7
	24 to 60	2,518	65,414	16.4	10.4
**Distal cytoplasmic droplets**	7 to 13	2,313	43,341	4.2	2.9
	14 to 23	1,980	76,398	4.2	2.9
	24 to 60	1,149	66,214	4.2	2.8
**Distal midpiece reflex**	7 to 13	2,312	43,131	2.9	3.0
	14 to 23	1,975	75,733	3.0	2.9
	24 to 60	1,149	65,572	3.1	2.8
**Bent tail**	7 to 13	2,300	40,187	0.9	0.7
	14 to 23	1,973	70,483	0.8	0.6
	24 to 60	1,143	60,950	0.8	0.6
**Abnormal head**	7 to 13	4,210	25,116	1.3	1.9
	14 to 23	3,952	46,686	1.0	1.5
	24 to 60	2,408	43,805	1.0	1.5

^1^All semen traits were measured with CASA systems, except abnormal head morphology which was assessed using standard microscopy. Semen traits of fresh semen were measured after pre-dilution. Mean and standard deviations are reported for untransformed traits.

### Variance components and genetic parameters

The estimates of phenotypic variance, heritabilities, and repeatabilities can be found in Table 2. In addition to the phenotypic variance, estimates of additive genetic, permanent environment, and residual variances can be found in Supplementary Table 3. The −log_10_(*P*-values) for pairwise comparisons between variance components at different ages are in Supplementary Table 6.

**Table 2.** Estimates of total phenotypic variance, heritability (*h*^*2*^) and repeatability (*rep*) for boars collected between 7 to 13 mo, 14 to 23 mo, and 24 to 60 mo of age from the trivariate analysis. Estimates are reported for untransformed semen quantity and transformed sperm motility and morphology traits

Trait	Age of the boar, mo	Phenotypic variance^1^	*h* ^*2*^	*rep*
**Semen quantity (untransformed)**				
Volume	7 to 13	9,344.2 ^(1.0)^	0.22 _(0.02)_	0.40 _(0.01)_
	14 to 23	11,625.0 ^(1.2)^	0.24 _(0.02)_	0.45 _(0.01)_
	24 to 60	13,854.0 ^(1.5)^	0.22 _(0.02)_	0.45 _(0.01)_
Concentration	7 to 13	4,277.6 ^(1.0)^	0.25 _(0.02)_	0.43 _(0.01)_
	14 to 23	5,126.7 ^(1.2)^	0.30 _(0.02)_	0.50 _(0.01)_
	24 to 60	5,611.8 ^(1.3)^	0.25 _(0.02)_	0.49 _(0.01)_
Total number of sperm cells	7 to 13	408.7 ^(1.0)^	0.22 _(0.02)_	0.44 _(0.01)_
	14 to 23	606.7 ^(1.5)^	0.23 _(0.02)_	0.46 _(0.01)_
	24 to 60	722.4 ^(1.8)^	0.20 _(0.02)_	0.46 _(0.01)_
**Sperm motility (transformed)**				
Total motility of fresh semen	7 to 13	152.9 ^(1.0)^	0.31 _(0.02)_	0.61 _(0.01)_
	14 to 23	158.2 ^(1.0)^	0.29 _(0.02)_	0.64 _(0.01)_
	24 to 60	171.0 ^(1.1)^	0.28 _(0.03)_	0.63 _(0.01)_
Total motility after 3 d of storage	7 to 13	300.8 ^(1.0)^	0.24 _(0.02)_	0.42 _(0.01)_
	14 to 23	284.2 ^(0.9)^	0.26 _(0.02)_	0.44 _(0.01)_
	24 to 60	293.9 ^(1.0)^	0.25 _(0.02)_	0.42 _(0.01)_
Progressive motility of fresh semen	7 to 13	231.3 ^(1.0)^	0.29 _(0.02)_	0.54 _(0.01)_
	14 to 23	244.5 ^(1.1)^	0.29 _(0.02)_	0.57 _(0.01)_
	24 to 60	265.3 ^(1.1)^	0.27 _(0.02)_	0.55 _(0.01)_
Progressive motility after 3 d of storage	7 to 13	235.0 ^(1.0)^	0.26 _(0.02)_	0.44 _(0.01)_
	14 to 23	225.5 ^(1.0)^	0.25 _(0.02)_	0.46 _(0.01)_
	24 to 60	233.7 ^(1.0)^	0.25 _(0.02)_	0.44 _(0.01)_
**Sperm morphology (transformed)**				
Total morphological abnormalities	7 to 13	4,315.9 ^(1.0)^	0.31 _(0.02)_	0.58 _(0.01)_
	14 to 23	4,707.9 ^(1.1)^	0.24 _(0.02)_	0.61 _(0.01)_
	24 to 60	4,968.7 ^(1.2)^	0.24 _(0.02)_	0.61 _(0.01)_
Distal cytoplasmic droplets	7 to 13	2,363.9 ^(1.0)^	0.26 _(0.03)_	0.53 _(0.01)_
	14 to 23	2,697.2 ^(1.1)^	0.31 _(0.03)_	0.59 _(0.01)_
	24 to 60	3,003.5 ^(1.3)^	0.28 _(0.04)_	0.60 _(0.01)_
Distal midpiece reflex	7 to 13	2,942.1 ^(1.0)^	0.28 _(0.03)_	0.65 _(0.01)_
	14 to 23	4,060.1 ^(1.4)^	0.31 _(0.04)_	0.74 _(0.01)_
	24 to 60	4,911.3 ^(1.7)^	0.30 _(0.04)_	0.76 _(0.01)_
Bent tail	7 to 13	1,000.8 ^(1.0)^	0.11 _(0.02)_	0.27 _(0.01)_
	14 to 23	960.4 ^(1.0)^	0.12 _(0.02)_	0.31 _(0.01)_
	24 to 60	983.1 ^(1.0)^	0.12 _(0.02)_	0.28 _(0.01)_
Abnormal head	7 to 13	3,992.3 ^(1.0)^	0.13 _(0.01)_	0.27 _(0.01)_
	14 to 23	3,538.3 ^(0.9)^	0.13 _(0.01)_	0.26 _(0.01)_
	24 to 60	3,544.6 ^(0.9)^	0.13 _(0.01)_	0.26 _(0.01)_

Standard errors are shown in subscript and were < 0.05 for heritability and < 0.02 for repeatability.

^1^Phenotypic variance was calculated based on the sum of additive genetic, permanent environment, herd-year-season of birth of the boar, collector-lab technician and residual variances. Phenotypic variance values estimated per age class are followed by their ratio, in superscript, calculated in reference to the phenotypic variance estimated at 7 to 13 mo.

For semen quantity traits, the phenotypic variance significantly increased with age class. Additive genetic, permanent environment, and residual variances also increased significantly with the age of the boar at collection. Consequently, the ratios of variances, i.e., heritability and repeatability, only changed to a small extent between ages.

For sperm motility traits, the phenotypic and residual variances significantly increased with age classes. The additive genetic variance of all motility traits did not significantly change with the age of the boar. The permanent environment variance significantly increases with age class for motility traits of fresh semen, but was not significantly different with age for motility traits after storage. Neither heritability nor repeatability estimates for sperm motility traits changed between ages.

For sperm morphology traits, phenotypic and residual variances increased with age, with the exception of phenotypic variance of bent tail. Increases in additive genetic variance were only observed for distal midpiece reflex between 7 to 13 and 14 to 23 mo (−log_10_(*P*-value) = 4.6) and between 7 to 13 and 24 to 60 mo (−log_10_(*P*-value) = 3.8). However, heritabilities for all sperm morphology traits were stable between age classes. Permanent environment variance significantly increased with age for total morphological abnormalities, distal cytoplasmic droplets, and distal midpiece reflex. Consequently, repeatability estimates increased for distal cytoplasmic droplets (i.e., 0.53 at 7 to 13 mo and 0.60 at 24 to 60 mo) and distal midpiece reflex (i.e., 0.65 at 7 to 13 mo and 0.76 at 24 to 60 mo).

### Genetic and permanent environment correlations

The additive genetic correlations between semen traits measured during the 3 ages are in Table 3. Estimates for phenotypic and permanent environment correlations, in addition to the additive genetic correlations, can be found in Supplementary Table 4.

**Table 3.** Additive genetic correlations between semen traits measured on boars collected between 7 to 13 mo, 14 to 23 mo, and 24 to 60 mo of age

Trait	7 to 13 mo and 14 to 23 mo	7 to 13 mo and 24 to 60 mo	14 to 23 mo and 24 to 60 mo
**Semen quantity**			
Volume, mL	0.95 _(0.01)_	0.89 _(0.03)_	0.97 _(0.01)_
Concentration, 10^6^/mL	0.95 _(0.01)_	0.89 _(0.02)_	0.95 _(0.01)_
Total number of sperm cells, 10^9^	0.93 _(0.02)_	0.89 _(0.03)_	0.96 _(0.01)_
**Sperm motility** ^1^			
Total motility of fresh semen	0.97 _(0.01)_	0.91 _(0.02)_	0.98 _(0.01)_
Total motility after 3 d of storage	0.90 _(0.02)_	0.81 _(0.04)_	0.94 _(0.02)_
Progressive motility of fresh semen	0.97 _(0.01)_	0.89 _(0.02)_	0.97 _(0.01)_
Progressive motility after 3 d of storage	0.91 _(0.02)_	0.82 _(0.04)_	0.95 _(0.02)_
**Sperm morphology** ^1^			
Total morphological abnormalities	0.97 _(0.01)_	0.85 _(0.03)_	0.95 _(0.02)_
Distal cytoplasmic droplets	0.97 _(0.01)_	0.93 _(0.03)_	0.98 _(0.01)_
Distal midpiece reflex	0.96 _(0.01)_	0.92 _(0.03)_	0.99 _(0.01)_
Bent tail	0.91 _(0.03)_	0.86 _(0.06)_	0.99 _(0.02)_
Abnormal head	0.97 _(0.02)_	0.95 _(0.03)_	0.99 _(0.01)_

Standard errors are shown in subscript and were < 0.05.

^1^Additive genetic correlations were reported for transformed traits. Estimates are reported for untransformed semen quantity and transformed sperm motility and morphology traits.

The additive genetic correlations for semen quantity traits were high between ages, all exceeding 0.89. For example, the genetic correlation of the total number of sperm cells between boars at 7 to 13 mo and 14 to 23 mo of age was 0.93; between boars at 14 to 23 mo and 24 to 60 mo of age was 0.96, and between boars at 7 to 13 mo and 24 to 60 mo of age was 0.89. We obtained similar estimates for ejaculate volume (*r*_*a*_ = 0.89 to 0.97) and concentration (*r*_*a*_ = 0.89 to 0.95).

For motility traits, the additive genetic correlations were also high between traits measured in different ages, with some differences between motility traits for fresh and stored semen. The genetic correlation between total motility of fresh semen measured at 7 to 13 mo of age and at 14 to 23 mo of age was 0.97; between 14 to 23 mo and 24 to 60 mo of age of the boar was 0.98, and between 7 to 13 mo and 24 to 60 mo of age was 0.91. These estimates were similar to the genetic correlations for progressive motility of fresh semen (*r*_*a*_ = 0.89 to 0.97).

For total motility after 3 d of storage, the genetic correlation between the trait measured at 7 to 13 mo and at 14 to 23 mo of age was 0.90, and between 14 to 23 mo and 24 to 60 mo of age was 0.94. However, the genetic correlation for the trait measured at 7 to 13 mo and at 24 to 60 mo of age of the boar was 0.81. These estimates were similar to those obtained for progressive motility after 3 d of storage (*r*_*a*_ = 0.82 to 0.95).

Finally, the genetic correlations for morphology traits measured at different collection periods were also high. For example, the additive genetic correlation of total morphological abnormalities measured at 7 to 13 mo and at 14 to 23 mo of age of the boar was 0.97, and between 14 to 23 mo and 24 to 60 mo of age of the boar was 0.95. The genetic correlation for the trait measured at 7 to 13 mo and at 24 to 60 mo of age of the boar was 0.85. These observations were consistent across all morphology traits (*r*_*a*_ = 0.93 to 0.98 for distal cytoplasmic droplets; *r*_*a*_ = 0.92 to 0.99 for distal midpiece reflex; *r*_*a*_ = 0.86 to 0.99 for bent tail, and *r*_*a*_ = 0.95 to 0.99 for abnormal head).

## Discussion

In this study, we examined whether the genetic parameters of semen traits changed with the age of the boar. We analyzed a large dataset with records on 12 semen traits from routine semen quality assessments in a commercial line. Culling of boars due to poor semen quality affected how averages for sperm motility and sperm morphology traits changed with the age of the boar. We divided the dataset into 3 age classes: 7 to 13 mo, 14 to 23 mo, and 24 to 60 mo of age. To account for possible effects of selection caused by culling of older boars due to poor semen quality, we used a trivariate approach to estimate variance components, heritabilities and repeatabilities, and genetic correlations between traits measured during the 3 different age classes. While variance components were heterogeneous, no large differences were observed for heritability at different ages. Additionally, we also observed high genetic correlations between semen traits measured at different ages.

### The impact of selection on age effects

We described how the number of service boars for semen collection changes with the age of the boar (Figure 1). Our results show a large decrease in the number of boars after an age of 15 to 17 mo, due to substantial culling. Other studies reported similar trends in the number of collected boars with age (Oh et al., 2006; Oh and See, 2008; Strathe et al., 2013). This suggests that the selection of boars with age is not unique to this population but a general condition. In fact, review studies on AI practices indicated that boar culling due to poor semen quality is considered a common practice (Robinson and Buhr, 2005; Knecht et al., 2017).

**Figure 3.**
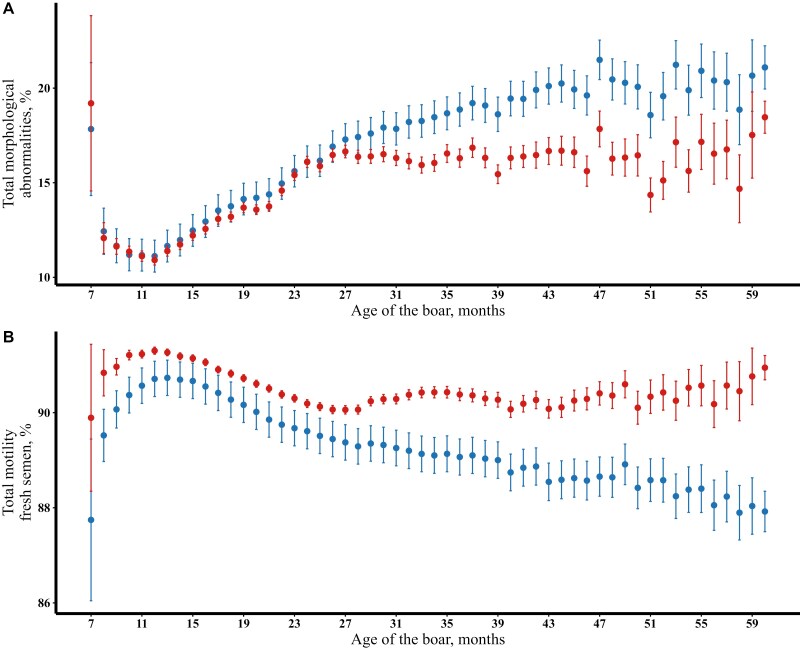
The effect of age of the boar at collection in a selected set of boars present in all age classes for (**A)** total morphological abnormalities (%) and (**B)** total motility of fresh semen (%). The blue markers represents the predicted effects based on model [1] and the red markers represents the unadjusted means of the trait. The error bars indicate a 95% confidence interval. Unadjusted means and estimated effects were reported for untransformed traits.

To the best of our knowledge, the impact of selection on estimates of systematic environmental factors in longitudinal traits has not been described. We advise to interpret these effects cautiously when such traits are under selection. In this study, we addressed the impact of culling due to poor semen quality on age effects in semen traits. For semen quantity traits, no large differences were observed between predicted and unadjusted trait means for the entire period of observation (Figure 2A), indicating that culling due to low semen quantity is not frequent in the population. For sperm motility traits, both predicted and unadjusted values increased until 13 mo, though unadjusted means were consistently higher than predicted values. After 13 mo, predicted values decreased while unadjusted means remained high (for example, Figure 2B). For sperm morphology traits, predicted and unadjusted means were similar until 11 mo but diverged thereafter, with predicted values increasing while unadjusted means remained low (Figure 2C). In addition, we also predicted the effect of the age of the boar on semen quality traits using records from a selected subset of 1,906 boars, which were collected in all 3 age classes (Figure 3). For this selected subset of boars, the raw averages of semen traits more closely matched the model-predicted means compared to those in the full dataset (Figure 2). Based on these observations, we concluded that the differences observed between predicted and unadjusted trait means with age reflect the impact of culling of older boars due to poor semen quality. When estimating the effect of age based on the full dataset, we expect the repeatability model to account for records from boars culled, whether due to poor genetics or unfavorable permanent environmental effects. As a result, this type of selection is accounted for in the analysis and predicted effects derived from the statistical analyses are closer to the true age effects than raw averages.

Culling of older boars due to poor semen quality can also affect estimates of genetic parameters when not properly accounted for. Based on the predicted effects, we divided the dataset into 3 age classes and estimated variance components following a trivariate approach. In this approach, records from the 3 age classes are fitted simultaneously and this should account for selection on semen traits, provided that the unselected trait—ejaculates collected between 7 and 13 mo—is included in the analysis (Meyer and Thompson, 1984; Walter and Mao, 1985; Meyer, 1991). To illustrate the effect of selection, we also estimated variance components in a univariate analysis (Supplementary Table 5), which cannot account for selection (Ayalew et al., 2017). Univariate analyses showed that traits under selection yield lower phenotypic and additive genetic variances as compared to multivariate analyses that account for selection (Rothschild et al., 1979; Meyer and Thompson, 1984; Visscher and Thompson, 1992). For semen quantity traits, the variance components estimated for the 3 ages were similar between the univariate and trivariate analyses, supporting the conclusion that culling due to low semen quantity is not frequent. For semen quality traits, the estimates of phenotypic and additive genetic variances at 14 to 23 mo and 24 to 60 mo were lower in the univariate analysis as compared to the trivariate analyses, consistent with what was expected. These observations indicate that culling due to poor semen quality affects estimates of genetic parameters at different ages when not properly accounted for in the analyses.

### The effect of age on semen traits

We investigated the effect of age on semen traits by predicting its effect on semen from boars of 7 to 60 mo of age (Supplementary Table 2). We observed trends that were consistent with reports from Wolf and Smital (2009) and Flowers (2022) in which the authors estimate the effect of age on semen traits with repeatability models in other pig populations. In our study, all boars were introduced into semen production after reaching puberty (i.e., around 7 mo of age). This is consistent with what is known about the development process of domestic pigs. Boars reach sexual maturity around 5 to 6 mo of age and experience puberty between 6 and 18 mo of age (Anderson, 2009). An increase in semen quantity during this period has been attributed to a continuous increase in testosterone and estradiol levels, which stimulate spermatogenesis and the production of seminal fluid in accessory glands (Anderson, 2009). In addition, other reports have described the importance of these hormones for epididymal function (Hess et al., 1997; Pearl et al., 2007), during which spermatozoa undergo later stages of maturation that confer the normal morphology and motility (Rodriguez-Martinez et al., 2009, 2022). Our observations on the increasing trend in semen quantity and sperm motility traits and the decreasing trend in morphological abnormalities between 7 and 13 mo of age coincide with the puberty period in a boar’s life and are likely explained by the increased hormonal response during this period.

### Variance components, genetic and permanent environment correlations

To study changes in the genetics of semen traits with age of the boar, we estimated variance components (for estimates, see Supplementary Table 3; for significance of pairwise differences, see Supplementary Table 6), heritabilities, and repeatabilities (Table 2) for age classes, and genetic correlations between age classes (Table 3). In addition, we also reported permanent environmental correlations (Supplementary Table 4). For semen quantity traits, permanent environment, additive genetic, and residual variances increased significantly, but changes in heritabilities and repeatabilities with age were small. For sperm motility, phenotypic and residual variances increase with age, but additive genetic variance was not significantly different between age classes. For sperm morphology, although additive genetic variance increased for some traits, heritability estimates remained stable with age. For all semen traits, genetic correlations between age classes were close to unity.

Our estimates of genetic correlations suggest that the same set of genetic variants influences these traits across different ages. The increase in additive genetic variance for semen quantity indicates that the effect of these genetic variants increases with age.

Our observations agreed with those of Strathe et al. (2013) but differed from the findings of Oh et al. (2006) and Hong et al. (2022). Both Oh’s and Hong’s teams fitted semen records using random regression models and described large oscillatory behavior of additive genetic variance with age of the boar and low to negative genetic correlation between semen traits measured at 33 wk (i.e., 8 mo of age) and at 150 wk (i.e., 37 mo of age; Oh et al., 2006; Hong et al., 2022). It is worth noting that both studies used high-order Legendre polynomials to describe changes in genetic and permanent environmental factors. It is generally considered that high-order polynomials generate waving patterns and are susceptible to “edge” effects, i.e., abrupt changes in estimated parameters around the edges (Meyer, 2005; Carabaño et al., 2007). Furthermore, it has also been pointed out that large oscillatory changes in the additive genetic variance of these traits with age are not biologically meaningful (Strathe et al., 2013). Strathe’s team also considered a random regression model to fit semen traits and while ignoring edges, the authors described minimal changes in heritability with age of the boar and high pairwise genetic correlations for boars collected between 40 wk (i.e., 10 mo) and 90 wk of age (i.e., 22 to 23 mo).

In this study, we divide our dataset into 3 age classes and model them using a trivariate analysis while accounting for repeated observations within each age class. Defining more age categories would significantly increase the number of parameters to estimate (Meyer and Hill, 1997) and may not be necessary if the trait variances are homogeneous and the genetic nature of the traits does not change within each age class. When using the repeatability model, we applied trait transformations to help normalize the trait distributions and stabilize their variances (Box and Cox, 1964). However, even under these conditions, we observed heterogeneous genetic variances for semen quantity and morphology traits and heterogeneous residual variances for all semen traits (Supplementary Table 6). Strathe et al. (2013) used a random regression model and Legendre polynomials to describe changes in genetic and permanent environmental factors, but assumed constant residual variances across ages, which is incompatible with the heterogeneous residual variances we report in our study.

Results from this study show that semen traits collected at different ages are genetically very similar traits, but the variances of most semen traits change with age. Therefore, a repeatability model in combination with data transformation might be a good way to analyze this type of data. Alternatively, a random regression model and Legendre polynomials can be used to describe changes in genetic and permanent environmental variance with age, while allowing residual variances to be different for specific age classes.

## Conclusions

The age of the boar has a considerable impact on porcine semen traits. Quantifying age effects based on unadjusted means may be misleading if boars are culled based on semen characteristics during their productive life, which is common in commercial settings. We estimated high genetic correlations between semen traits measured at different ages, indicating that the same genetic variants influence these traits throughout the production period. The genetic variance remained stable for semen quality traits with age class but increased for semen quantity, indicating that the effect of genetic variants affecting semen quantity traits increases with the age of the boar.

## Supplementary Material

skaf257_suppl_Supplementary_Table_S1

skaf257_suppl_Supplementary_Table_S2

skaf257_suppl_Supplementary_Table_S3

skaf257_suppl_Supplementary_Table_S4

skaf257_suppl_Supplementary_Table_S5

skaf257_suppl_Supplementary_Table_S6

## Abbreviations

CASA: computer-assisted sperm analysis
GWAS: genome-wide association studies
AI: artificial insemination
