# Supplementary material for: The effect of aging on genetic parameters of boar semen traits
Source: J Anim Sci. 2025 Aug 1;103:skaf257. doi: 10.1093/jas/skaf257 (PMC12445636; doi:10.1093/jas/skaf257)
Supplement: skaf257_suppl_Supplementary_Table_S1 [file skaf257_suppl_supplementary_table_s1.docx]

**Supplementary Table 1.** Description of semen records of boars from a commercial line.

| **Trait^1)^** | **Number of Boars** | **Observations** | **Mean** | **SD** | **CV** |
| --- | --- | --- | --- | --- | --- |
| **Semen Quantity** | | | | |  |
| **Volume (mL)** | 5,634 | 449,202 | 391.0 | 121.4 | 3.2 |
| **Concentration (10^6^/mL)** | 5,633 | 448,632 | 188.0 | 76.6 | 2.5 |
| **Total number of sperm cells (x10^9^)** | 5,634 | 449,361 | 70.4 | 26.9 | 2.6 |
| **Sperm Motility (%)** | | | | |  |
| **Total motility of fresh semen** | 5,599 | 425,885 | 90.1 | 4.8 | 18.8 |
| **Total motility after 3 days of storage** | 4,784 | 190,319 | 79.7 | 10.6 | 7.5 |
| **Progressive motility of fresh semen** | 5,631 | 445,745 | 80.9 | 8.2 | 9.9 |
| **Progressive motility after 3 days of storage** | 4,785 | 190,533 | 70.9 | 11.4 | 6.2 |
| **Sperm Morphology (%)** | | | | |  |
| **Total morphological abnormalities** | 5,477 | 182,657 | 15.9 | 10.7 | 1.5 |
| **Distal cytoplasmatic droplets** | 2,736 | 186,540 | 4.2 | 2.9 | 1.4 |
| **Distal Midpiece Reflex** | 2,736 | 185,022 | 3.0 | 2.9 | 1.0 |
| **Bent Tail** | 2,734 | 172,224 | 0.8 | 0.7 | 1.1 |
| **Abnormal Head** | 5,229 | 116,941 | 1.0 | 1.6 | 0.6 |

^1)^All semen traits were measured with CASA systems, except abnormal head morphology which was measured using standard microscopy assessments. SD = standard deviation; CV = Coefficent of varaition. Means and standard deviations are reported for untransformed traits. Semen traits of fresh semen were measured after pre-dilution.
