# Supplementary material for: The effect of aging on genetic parameters of boar semen traits
Source: J Anim Sci. 2025 Aug 1;103:skaf257. doi: 10.1093/jas/skaf257 (PMC12445636; doi:10.1093/jas/skaf257)
Supplement: skaf257_suppl_Supplementary_Table_S3 [file skaf257_suppl_supplementary_table_s3.docx]

**Supplementary Table 3.** Estimates of phenotypic, additive genetics, permanent environment and residual variances, for semen traits measured at 7-13 months, 14-23 months and 24-60 months of age of the boar in a trivariate analysis. Estimates are reported for untransformed semen quantity and transformed sperm motility and morphology traits.

| **Trait** | **Age of the boar** | **Phenotypic**  **Variance^1)^** | **Additive Genetic Variance** | **Permanent Environment Variance** | **Residual Variance** |
| --- | --- | --- | --- | --- | --- |
| **Semen Quantity (Untransformed)** |  |  |  |  |  |
| **Volume** | 7-13 | 9,344.2 _(121.4)_ | 2,088.8 _(174.5)_ | 1,656.1 _(113.6)_ | 4,700.2 _(22.7)_ |
|  | 14-23 | 11,625.0 _(147.9)_ | 2,788.7 _(229.3)_ | 2,470.3 _(151.6)_ | 5,510.9 _(18.3)_ |
|  | 24-60 | 13,854.0 _(197.7)_ | 3,114.8 _(291.6)_ | 3,052.0 _(207.3)_ | 6,394.6 _(22.2)_ |
| **Concentration** | 7-13 | 4,277.6 _(55.7)_ | 1,074.0 _(83.5)_ | 746.0 _(53.3)_ | 2,154.5 _(10.4)_ |
|  | 14-23 | 5,126.7 _(71.0)_ | 1,528.6 _(113.5)_ | 1,031.7 _(71.7)_ | 2,314.9 _(7.7)_ |
|  | 24-60 | 5,611.8 _(80.8)_ | 1,406.9 _(120.4)_ | 1,356.9 _(86.0)_ | 2,565.1 _(8.9)_ |
| **Total number of sperm cells** | 7-13 | 408.7 _(5.1)_ | 91.4 _(8.2)_ | 88.0 _(5.5)_ | 216.8 _(1.0)_ |
|  | 14-23 | 606.7 _(7.6)_ | 137.5 _(12.1)_ | 143.6 _(8.3)_ | 309.3 _(1.0)_ |
|  | 24-60 | 722.4 _(9.9)_ | 147.4 _(15.0)_ | 186.3 _(11.3)_ | 369.8 _(1.3)_ |
| **Sperm Motility (Transformed)** |  |  |  |  |  |
| **Total motility of fresh semen** | 7-13 | 152.9 _(2.5)_ | 46.6 _(4.2)_ | 46.9 _(2.8)_ | 56.7 _(0.3)_ |
|  | 14-23 | 158.2 _(2.7)_ | 46.5 _(4.3)_ | 54.7 _(3.0)_ | 55.7 _(0.2)_ |
|  | 24-60 | 171.0 _(3.3)_ | 47.6 _(4.8)_ | 60.5 _(3.6)_ | 60.3 _(0.2)_ |
| **Total motility after 3 days of storage** | 7-13 | 300.8 _(4.7)_ | 72.9 _(6.7)_ | 52.4 _(4.5)_ | 157.9 _(1.2)_ |
|  | 14-23 | 284.2 _(4.0)_ | 74.4 _(6.1)_ | 50.7 _(4.0)_ | 150.1 _(0.8)_ |
|  | 24-60 | 293.9 _(4.6)_ | 73.6 _(6.9)_ | 49.0 _(4.7)_ | 161.1 _(0.9)_ |
| **Progressive motility of fresh semen** | 7-13 | 231.3 _(3.4)_ | 67.6 _(5.6)_ | 56.7 _(3.6)_ | 100.7 _(0.5)_ |
|  | 14-23 | 244.5 _(3.7)_ | 71.1 _(5.9)_ | 68.1 _(4.0)_ | 101.1 _(0.3)_ |
|  | 24-60 | 265.3 _(4.6)_ | 71.1 _(6.7)_ | 59.8 _(4.0)_ | 109.8 _(0.4)_ |
| **Progressive motility after 3 days of storage** | 7-13 | 235.0 _(3.5)_ | 61.0 _(5.6)_ | 43.0 _(3.7)_ | 123.3 _(0.9)_ |
|  | 14-23 | 225.5 _(3.1)_ | 57.0 _(4.9)_ | 46.0 _(3.3)_ | 118.8 _(0.6)_ |
|  | 24-60 | 233.7 _(3.7)_ | 54.8 _(5.5)_ | 48.7 _(3.9)_ | 125.7 _(0.7)_ |
| **Sperm Morphology (Transformed)** |  |  |  |  |  |
| **Total morphological abnormalities** | 7-13 | 4,315.9 _(73.9)_ | 1,317.9 _(118.3)_ | 1,199.4 _(79.9)_ | 1,660.8 _(11.9)_ |
|  | 14-23 | 4,707.9 _(77.9)_ | 1,142.9 _(112.5)_ | 1,743.5 _(84.3)_ | 1,728.4 _(9.4)_ |
|  | 24-60 | 4,968.7 _(98.0)_ | 1,206.5 _(139.2)_ | 1,831.5 _(108.9)_ | 1,836.7 _(10.4)_ |
| **Distal cytoplasmic droplets** | 7-13 | 2,363.9 _(49.0)_ | 623.3 _(78.2)_ | 634.4 _(52.6)_ | 1,032.5 _(7.2)_ |
|  | 14-23 | 2,697.2 _(62.3)_ | 842.8 _(102.9)_ | 751.2 _(67.9)_ | 1,041.2 _(5.4)_ |
|  | 24-60 | 3,003.5 _(79.0)_ | 846.1 _(120.2)_ | 954.3 _(87.6)_ | 1,135.4 _(6.3)_ |
| **Distal midpiece reflex** | 7-13 | 2,942.1 _(68.7)_ | 817.8 _(113.6)_ | 1,100.4 _(80.0)_ | 1,003.7 _(7.1)_ |
|  | 14-23 | 4,060.1 _(108.1)_ | 1,260.4 _(171.3)_ | 1,743.3 _(122.4)_ | 1,045.0 _(5.5)_ |
|  | 24-60 | 4,911.3 _(150.7)_ | 1,461.1 _(220.0)_ | 2,262.3 _(169.6)_ | 1,159.9 _(6.5)_ |
| **Bent tail** | 7-13 | 1,000.8 _(12.8)_ | 110.9 _(18.5)_ | 164.0 _(13.8)_ | 706.2 _(5.2)_ |
|  | 14-23 | 960.4 _(12.6)_ | 119.3 _(18.6)_ | 176.7 _(13.9)_ | 652.1 _(3.5)_ |
|  | 24-60 | 983.1 _(14.7)_ | 113.6 _(19.3)_ | 164.3 _(15.3)_ | 686.9 _(4.0)_ |
| **Abnormal head** | 7-13 | 3,992.3 _(54.9)_ | 509.9 _(56.3)_ | 552.3 _(44.7)_ | 2,736.3 _(26.7)_ |
|  | 14-23 | 3,538.3 _(39.0)_ | 455.3 _(44.2)_ | 468.4 _(32.9)_ | 2,507.2 _(17.1)_ |
|  | 24-60 | 3,544.6 _(44.7)_ | 443.1 _(48.9)_ | - 1. _(38.4)_ | 2,467.2 _(17.1)_ |

Standard errors of the estimates are shown in subscript. 1) Phenotypic variance was calculated based on the sum of additive genetic, permanent environment, herd-year-season of birth of the boar, collector-lab technician and residual variances.
