# Supplementary material for: The effect of aging on genetic parameters of boar semen traits
Source: J Anim Sci. 2025 Aug 1;103:skaf257. doi: 10.1093/jas/skaf257 (PMC12445636; doi:10.1093/jas/skaf257)
Supplement: skaf257_suppl_Supplementary_Table_S4 [file skaf257_suppl_supplementary_table_s4.docx]

**Supplementary Table 4.** Phenotypic (*r^p^*), additive genetic (*r^a^*) and permanent environment (*r^pe^*) correlations for semen traits from boars collected between 7 – 13 months, 14 – 23 months and 24 – 60 months of age.

| **Trait** | **Age of the boar** | ***r^p^*** | ***r^a^*** | ***r^pe^*** |
| --- | --- | --- | --- | --- |
| **Semen Quantity** |  |  |  |  |
| Volume | 7-13 & 14-23 | 0.37 _(0.01)_ | 0.95 _(0.01)_ | 0.78 _(0.02)_ |
|  | 7-13 & 24-60 | 0.44 _(0.01)_ | 0.89 _(0.03)_ | 0.58 _(0.03)_ |
|  | 14-23 & 24-60 | 0.28 _(0.01)_ | 0.97 _(0.01)_ | 0.77 _(0.02)_ |
| Concentration | 7-13 & 14-23 | 0.40 _(0.01)_ | 0.95 _(0.01)_ | 0.76 _(0.02)_ |
|  | 7-13 & 24-60 | 0.48 _(0.01)_ | 0.89 _(0.02)_ | 0.60 _(0.03)_ |
|  | 14-23 & 24-60 | 0.32 _(0.01)_ | 0.95 _(0.01)_ | 0.82 _(0.02)_ |
| Total number of sperm cells | 7-13 & 14-23 | 0.37 _(0.01)_ | 0.93 _(0.02)_ | 0.72 _(0.02)_ |
|  | 7-13 & 24-60 | 0.50 _(0.01)_ | 0.89 _(0.03)_ | 0.53 _(0.03)_ |
|  | 14-23 & 24-60 | 0.26 _(0.01)_ | 0.96 _(0.01)_ | 0.81 _(0.02)_ |
| **Sperm Motility** |  |  |  |  |
| Total motility of fresh semen | 7-13 & 14-23 | 0.57 _(0.01)_ | 0.97 _(0.01)_ | 0.87 _(0.01)_ |
|  | 7-13 & 24-60 | 0.60 _(0.01)_ | 0.91 _(0.02)_ | 0.75 _(0.02)_ |
|  | 14-23 & 24-60 | 0.51 _(0.01)_ | 0.98 _(0.01)_ | 0.89 _(0.01)_ |
| Total motility after 3 days of storage | 7-13 & 14-23 | 0.35 _(0.01)_ | 0.90 _(0.02)_ | 0.68 _(0.03)_ |
|  | 7-13 & 24-60 | 0.35 _(0.01)_ | 0.81 _(0.04)_ | 0.49 _(0.05)_ |
|  | 14-23 & 24-60 | 0.29 _(0.01)_ | 0.94 _(0.02)_ | 0.69 _(0.03)_ |
| Progressive motility of fresh semen | 7-13 & 14-23 | 0.48 _(0.01)_ | 0.97 _(0.01)_ | 0.76 _(0.02)_ |
|  | 7-13 & 24-60 | 0.52 _(0.01)_ | 0.89 _(0.02)_ | 0.59 _(0.03)_ |
|  | 14-23 & 24-60 | 0.39 _(0.01)_ | 0.97 _(0.01)_ | 0.83 _(0.01)_ |
| Progressive motility after 3 days of storage | 7-13 & 14-23 | 0.37 _(0.01)_ | 0.91 _(0.02)_ | 0.68 _(0.03)_ |
|  | 7-13 & 24-60 | 0.38 _(0.01)_ | 0.82 _(0.04)_ | 0.50 _(0.05)_ |
|  | 14-23 & 24-60 | 0.31 _(0.01)_ | 0.95 _(0.02)_ | 0.75 _(0.02)_ |
| **Sperm Morphology** |  |  |  |  |
| Total morphological abnormalities | 7-13 & 14-23 | 0.51 _(0.01)_ | 0.97 _(0.01)_ | 0.77 _(0.02)_ |
|  | 7-13 & 24-60 | 0.56 _(0.01)_ | 0.85 _(0.03)_ | 0.65 _(0.03)_ |
|  | 14-23 & 24-60 | 0.42 _(0.01)_ | 0.95 _(0.02)_ | 0.83 _(0.01)_ |
| Distal cytoplasmic droplets | 7-13 & 14-23 | 0.50 _(0.01)_ | 0.97 _(0.01)_ | 0.81 _(0.02)_ |
|  | 7-13 & 24-60 | 0.59 _(0.01)_ | 0.93 _(0.03)_ | 0.68 _(0.03)_ |
|  | 14-23 & 24-60 | 0.42 _(0.01)_ | 0.98 _(0.01)_ | 0.87 _(0.02)_ |
| Distal midpiece reflex | 7-13 & 14-23 | 0.63 _(0.01)_ | 0.96 _(0.01)_ | 0.87 _(0.01)_ |
|  | 7-13 & 24-60 | 0.83 _(0.01)_ | 0.92 _(0.03)_ | 0.77 _(0.02)_ |
|  | 14-23 & 24-60 | 0.50 _(0.01)_ | 0.99 _(0.01)_ | 0.92 _(0.01)_ |
| Bent tail | 7-13 & 14-23 | 0.24 _(0.01)_ | 0.91 _(0.03)_ | 0.77 _(0.03)_ |
|  | 7-13 & 24-60 | 0.25 _(0.01)_ | 0.86 _(0.06)_ | 0.60 _(0.05)_ |
|  | 14-23 & 24-60 | 0.20 _(0.01)_ | 0.99 _(0.02)_ | 0.79 _(0.03)_ |
| Abnormal Head | 7-13 & 14-23 | 0.24 _(0.01)_ | 0.97 _(0.02)_ | 0.87 _(0.03)_ |
|  | 7-13 & 24-60 | 0.23 _(0.01)_ | 0.95 _(0.03)_ | 0.76 _(0.04)_ |
|  | 14-23 & 24-60 | 0.24 _(0.01)_ | 0.99 _(0.01)_ | 0.88 _(0.02)_ |

Standard errors are shown in subscript and were < 0.06. Estimates are reported for untransformed semen quantity and transformed sperm motility and morphology traits.
