# Supplementary material for: The effect of aging on genetic parameters of boar semen traits
Source: J Anim Sci. 2025 Aug 1;103:skaf257. doi: 10.1093/jas/skaf257 (PMC12445636; doi:10.1093/jas/skaf257)
Supplement: skaf257_suppl_Supplementary_Table_S5 [file skaf257_suppl_supplementary_table_s5.docx]

**Supplementary Table 5.** Estimates of total phenotypic, additive genetics and permanent environment variances, for boars collected between 7 – 13 months, 14 – 23 months, and 24 – 60 months of age from the univariate analysis.

| **Trait** | **Age of the boar** | **Phenotypic**  **Variance^1)^** | **Additive Genetic Variance** | **Permanent Environment Variance** |
| --- | --- | --- | --- | --- |
| **Semen Quantity (Untransformed)** |  |  |  |  |
| **Volume** | 7-13 months | 9,377.7 _(148.9)_ | 1,778.5 _(168.4)_ | 1,590.9 _(111.1)_ |
|  | 14-23 months | 11,484 _(164.7)_ | 2,481.1 _(234.7)_ | 2,267.9 _(154.2)_ |
|  | 24-60 months | 14,023 _(238.9)_ | 3,212.9 _(383.9)_ | 2,859.3 _(265.5)_ |
| **Concentration** | 7-13 months | 4,150.7 _(62.9)_ | 813.3 _(74.2)_ | 703.1 _(48.9)_ |
|  | 14-23 months | 5,055.5 _(81.4)_ | 1,461.7 _(115.5)_ | 823.9 _(70.5)_ |
|  | 24-60 months | 5,711.4 _(98.8)_ | 1,616.7 _(163.3)_ | 1,186.6 _(110.4)_ |
| **Total number of sperm cells** | 7-13 months | 408.8 _(5.3)_ | 91.5 _(8.6)_ | 87.0 _(5.7)_ |
|  | 14-23 months | 592.0 _(7.7)_ | 134.0 _(12.7)_ | 129.1 _(8.5)_ |
|  | 24-60 months | 705.6 _(11.0)_ | 160.5 _(19.1)_ | 154.9 _(13.5)_ |
| **Sperm Motility (Transformed)** |  |  |  |  |
| **Total motility of fresh semen** | 7-13 months | 156.4 _(2.8)_ | 42.4 _(4.4)_ | 49.5 _(3.0)_ |
|  | 14-23 months | 120.8 _(1.8)_ | 26.8 _(2.9)_ | 33.9 _(2.0)_ |
|  | 24-60 months | 117.2 _(1.9)_ | 20.5 _(3.0)_ | 31.1 _(2.3)_ |
| **Total motility after 3 days of storage** | 7-13 months | 306.5 _(5.4)_ | 76.5 _(7.5)_ | 50.2 _(4.9)_ |
|  | 14-23 months | 276.5 _(4.2)_ | 66.3 _(6.1)_ | 44.9 _(3.9)_ |
|  | 24-60 months | 276.7 _(4.7)_ | 57.1 _(6.7)_ | 39.2 _(4.5)_ |
| **Progressive motility of fresh semen** | 7-13 months | 237.3 _(4.0)_ | 60.0 _(5.8)_ | 61.4 _(3.9)_ |
|  | 14-23 months | 212.3 _(3.1)_ | 49.7 _(5.0)_ | 52.9 _(3.3)_ |
|  | 24-60 months | 209.1 _(3.2)_ | 38.6 _(5.2)_ | 47.6 _(3.8)_ |
| **Progressive motility after 3 days of storage** | 7-13 months | 238.6 _(3.9)_ | 62.6 _(6.1)_ | 41.8 _(4.0)_ |
|  | 14-23 months | 219.9 _(3.2)_ | 52.9 _(5.1)_ | 41.0 _(3.4)_ |
|  | 24-60 months | 219.4 _(3.6)_ | 45.1 _(5.6)_ | 39.1 _(3.9)_ |
| **Sperm Morphology (Transformed)** |  |  |  |  |
| **Total morphological abnormalities** | 7-13 months | 4,407.0 _(83.6)_ | 1,142.2 _(122.7)_ | 1,282.1 _(84.8)_ |
|  | 14-23 months | 4,276.7 _(69.6)_ | 741.0 _(101.8)_ | 1,627.4 _(79.6)_ |
|  | 24-60 months | 3,980.5 _(72.3)_ | 580.3 _(113.1)_ | 1,410.2 _(93.5)_ |
| **Distal cytoplasmic droplets** | 7-13 months | 2,389.5 _(58.2)_ | 473.9 _(78.3)_ | 712.5 _(56.3)_ |
|  | 14-23 months | 2,488.0 _(61.0)_ | 664.4 _(101.1)_ | 656.0 _(67.5)_ |
|  | 24-60 months | 2,531.4 _(72.0)_ | 675.5 _(130.7)_ | 622.9 _(90.8)_ |
| **Distal midpiece reflex** | 7-13 months | 2,975.1 _(73.9)_ | 744.8 _(126.0)_ | 1,164.7 _(89.9)_ |
|  | 14-23 months | 3,413.5 _(90.1)_ | 905.5 _(146.5)_ | 1,442.4 _(107.2)_ |
|  | 24-60 months | 3,304.1 _(104.0)_ | 787.9 _(175.1)_ | 1,300.7 _(134.5)_ |
| **Bent tail** | 7-13 months | 1,005.6 _(13.5)_ | 92.5 _(18.7)_ | 181.4 _(14.7)_ |
|  | 14-23 months | 908.1 _(10.9)_ | 80.2 _(15.7)_ | 157.1 _(12.2)_ |
|  | 24-60 months | 909.6 _(12.0)_ | 74.9 _(17.4)_ | 130.2 _(14.0)_ |
| **Abnormal head** | 7-13 months | 4,000.7 _(59.1)_ | 485.4 _(62.0)_ | 506.2 _(47.6)_ |
|  | 14-23 months | 3,468.9 _(39.6)_ | 378.2 _(44.2)_ | 434.4 _(33.1)_ |
|  | 24-60 months | 3,480.1 _(47.2)_ | 412.9 _(58.1)_ | 413.1 _(43.5)_ |

Phenotypic variance was calculated based on the sum of additive genetic, permanent environment, herd-year-season of birth of the boar, collector-lab technician and residual variances. Estimates are reported for untransformed semen quantity and transformed sperm motility and morphology traits.
