# Supplementary material for: The effect of aging on genetic parameters of boar semen traits
Source: J Anim Sci. 2025 Aug 1;103:skaf257. doi: 10.1093/jas/skaf257 (PMC12445636; doi:10.1093/jas/skaf257)
Supplement: skaf257_suppl_Supplementary_Table_S6 [file skaf257_suppl_supplementary_table_s6.docx]

**Supplementary Table 6.** The -log_10_(*P*-values) for the significance of the differences between variance components estimated at 7-13 months, 14-23 months and 24-60 months of age of the boar in a trivariate analysis.

| **Trait** | **Age of the boar** | **Phenotypic**  **Variance** | **Additive Genetic Variance** | **Permanent Environment Variance** | **Residual Variance** |
| --- | --- | --- | --- | --- | --- |
| **Semen Quantity (Untransformed)** |  |  |  |  |  |
| **Volume** | 7-13 & 14-23 months | **67.2** | **5.0** | **11.2** | **169.5** |
|  | 7-13 & 24-60 months | **118.1** | **4.1** | **11.9** | **300.0** |
|  | 14-23 & 24-60 months | **39.9** | 1.0 | 3.4 | **206.3** |
| **Concentration** | 7-13 & 14-23 months | **46.8** | **7.9** | **6.3** | **34.6** |
|  | 7-13 & 24-60 months | **64.6** | 2.7 | **12.9** | **196.9** |
|  | 14-23 & 24-60 months | **12.6** | 0.8 | **5.4** | **99.4** |
| **Total number of sperm cells** | 7-13 & 14-23 months | **216.3** | **6.3** | **15.2** | **300.0** |
|  | 7-13 & 24-60 months | **247.1** | **4.6** | **19.2** | **300.0** |
|  | 14-23 & 24-60 months | **48.3** | 0.5 | **5.6** | **294.8** |
| **Sperm Motility (Transformed)** |  |  |  |  |  |
| **Total motility of fresh semen** | 7-13 & 14-23 months | 2.3 | 0.1 | **3.8** | 2.4 |
|  | 7-13 & 24-60 months | **9.2** | 0.1 | **4.8** | **24.5** |
|  | 14-23 & 24-60 months | **7.8** | 0.2 | 1.9 | **57.7** |
| **Total motility after 3 days of storage** | 7-13 & 14-23 months | 3.3 | 0.1 | 0.2 | **7.2** |
|  | 7-13 & 24-60 months | 0.6 | 0.1 | 0.3 | 1.6 |
|  | 14-23 & 24-60 months | 1.6 | 0.1 | 0.2 | **20.6** |
| **Progressive motility of fresh semen** | 7-13 & 14-23 months | **5.1** | 0.4 | **3.6** | 0.3 |
|  | 7-13 & 24-60 months | **13.5** | 0.2 | **4.4** | **48.5** |
|  | 14-23 & 24-60 months | **8.4** | 0.1 | 1.5 | **65.0** |
| **Progressive motility after 3 days of storage** | 7-13 & 14-23 months | 2.3 | 0.4 | 0.4 | **4.2** |
|  | 7-13 & 24-60 months | 0.1 | 0.5 | 0.6 | 1.4 |
|  | 14-23 & 24-60 months | 1.9 | 0.2 | 0.3 | **13.1** |
| **Sperm Morphology (Transformed)** |  |  |  |  |  |
| **Total morphological abnormalities** | 7-13 & 14-23 months | **7.4** | 1.3 | **12.6** | **5.1** |
|  | 7-13 & 24-60 months | **9.9** | 0.4 | **7.9** | **28.1** |
|  | 14-23 & 24-60 months | 2.7 | 0.3 | 0.5 | **14.0** |
| **Distal cytoplasmic droplets** | 7-13 & 14-23 months | **12.2** | 3.0 | 1.7 | 0.5 |
|  | 7-13 & 24-60 months | **18.4** | 1.6 | **4.2** | **26.0** |
|  | 14-23 & 24-60 months | **6.9** | 0.1 | 2.9 | **29.0** |
| **Distal midpiece reflex** | 7-13 & 14-23 months | **50.5** | **4.6** | **13.9** | **5.4** |
|  | 7-13 & 24-60 months | **52.4** | **3.8** | **15.3** | **59.0** |
|  | 14-23 & 24-60 months | **18.1** | 1.1 | **6.1** | **41.1** |
| **Bent tail** | 7-13 & 14-23 months | 2.5 | 0.2 | 0.4 | **17.3** |
|  | 7-13 & 24-60 months | 0.5 | 0.1 | 0.1 | 2.5 |
|  | 14-23 & 24-60 months | 1.0 | 0.1 | 0.4 | **10.2** |
| **Abnormal head** | 7-13 & 14-23 months | **13.8** | 0.7 | 1.3 | **12.3** |
|  | 7-13 & 24-60 months | **11.4** | 0.7 | 0.7 | **16.7** |
|  | 14-23 & 24-60 months | 0.1 | 0.1 | 0.2 | 1.0 |

The difference between two variance components was significant when -log_10_(*P*-value) > 3.5. Significant differences are in bold.
